# Maternal SO_2_ exposure and birthweight reduction during COVID-19 lockdown and quarantine in East China

**DOI:** 10.3389/fendo.2026.1954694

**Published:** 2026-09-18

**Authors:** Rui Feng, Fengbing Liang, Xinhui Wang, Jiandong Shen, Cuiyu Yang

**Affiliations:** 1 School of Engineering, Hangzhou Normal University, Hangzhou, China; 2 Department of Obstetrics and Gynecology, Sir Run Run Shaw Hospital, School of Medicine, Zhejiang University, Hangzhou, China; 3 Zhejiang Key Laboratory of Precise Protection and Promotion of Fertility, Hangzhou, China; 4 Zhejiang Provincial Clinical Research Center for Reproductive Health and Disease, Hangzhou, China; 5 School of Public Health, Zhejiang University School of Medicine, Hangzhou, China; 6 Hangzhou Environmental Monitoring Central Station, Hangzhou, China

**Keywords:** air pollution, birthweight prediction, COVID-19 lockdown, machine learning, odds ratio, sulfur dioxide

## Abstract

**Background:**

Low birthweight remains a global public health challenge. While maternal exposure to certain air pollutants is known to adversely affect fetal growth, the association with sulfur dioxide (SO_2_) has been inconsistent. The COVID-19 lockdown provided a unique quasi-natural experiment to reassess the relationship under altered emission patterns.

**Methods:**

We conducted a retrospective cohort study of 21, 777 term singleton births in Southern Zhejiang Province, East China, from 2020 to 2022. We estimated maternal SO_2_ exposure using a time-activity weighted model based on residential and work addresses. Associations with low and high birthweight risks were evaluated using linear regression and odds ratios. Three machine learning models were used to assess whether incorporating SO_2_ exposure improved birthweight prediction accuracy.

**Results:**

The COVID-19 lockdown was associated with a significant 28% reduction in annual mean atmospheric SO_2_ levels. Maternal SO_2_ exposure during the third trimester showed the most substantial negative association with birthweight, particularly in female neonates, and was associated with increased odds of LBW in both sexes. Feature importance showed SO_2_ played a modest but nonnegligible role. Adding SO_2_ exposure as a predictor improved birthweight prediction, with XGBoost achieving the highest accuracy.

**Conclusion:**

We found the susceptible windows of maternal SO_2_ exposure for birthweight reduction and LBW risks. Incorporating air pollution exposure into predictive models can enhance the identification of high-risk pregnancies, supporting targeted clinical and environmental interventions.

## 1 Introduction

Low birthweight (LBW) (less than 2.5 kg) is associated with a variety of adverse reproductive outcomes, including increased neonatal mortality, growth and developmental retardation, and a higher risk of chronic diseases including hypertension, cardiovascular disease, and type 2 diabetes in adulthood (1). In 2020, an estimated 19.8 (95% confidence interval (CI): 18.4–21.7) million neonates were born with LBW around the world, accounting for 14.7% (95% CI 13.7–16.1) of all liveborn neonates globally (2). Despite reduction in LBW prevalence (2), LBW has become a cause of major loss in human capital for decades, leading to long-term adverse impacts on neonates, their parents, and societies (3). Globally, approximately 80% of all neonatal deaths were ascribed to LBW (3). Small-for-gestational-age (SGA) infants have poorer health than those with normal birthweight (4) (5). Infants with high birthweight (HBW; macrosomia; more than 4.0 kg) (6) and large for gestational age (LGA) (7) are associated with a higher risk of neonatal diseases as well.

Maternal exposure to air pollution during pregnancy (8) or in preconception period (9) is associated with adverse birth outcomes, including lower fetal growth (8), LBW, and preterm birth (PTB) (10, 11). In 2005, the prevalence of LBW infants in the United States was around 8.2% (12). Reportedly, decreased birthweight was associated with maternal exposure to carbon monoxide (CO) (13), nitrogen dioxide (NO_2_) (14, 15), NOx (16), particulate matter less than 10 and 2.5 microns (PM_10_ and PM_2.5_) (12, 17), ground-level ozone (O_3_) (18, 19), and black carbon (20, 21). For per interquartile range (IQR) increase in PM_2.5_ during the second trimester, birthweight decreased by 21.1 (95% confidence interval (CI): 0.4-41.8) g in Seoul (22). With per 10 μg/m^3^ increment in ambient PM_2.5_, mean birthweight reduced by 22 g and risk of LBW increased by 11% (23). In low- and middle-income countries (e.g., South Asia and sub-Saharan Africa), for every 10–part per billion (ppb) increment in ambient O_3_ level, birthweight reduced by 19.9 (95% CI: 14.8 to 24.9) g, with average decrease in birthweight of 43.8 (95% CI: 30.5 to 54.3) g in 2019 (19). In the United States, with 10 ppb increase in ambient O_3_ concentration during the entire pregnancy, term birthweight reduced by 7.6 (95% CI: 6.4 to 8.8) g (24). Susceptible windows were identified (9, 17, 18).

However, previous studies showed less consistent and heterogeneities for linking birthweight with maternal exposure to sulfur dioxide (SO_2_) (9, 12, 14, 25). The sudden slowdown of human activities during the COVID-19 lockdown and quarantine caused changes in air pollution emission patterns including SO_2_ (26), offering a rare opportunity for scientific research that is normally difficult to obtain. The large-scale, synchronized socioeconomic standstill created an unprecedented “quasi-natural experimental” scenario. Researchers were thus able to observe, in the real world, how air quality and public health would respond rapidly when major anthropogenic emission sources—such as traffic and industry—were substantially cut off.

This study explored the association of maternal SO_2_ exposure during pregnancy and neonatal birthweight in eastern cities of Zhejiang Province during the COVID-19 lockdown. We assessed the impacts of maternal SO_2_ exposure on the occurrence of LBW and HBW infants, and evaluated the improvement in accuracy of predicting birthweight using machine learning models with adding maternal SO_2_ exposure as input parameters.

## 2 Methods

### 2.1 Population

The investigated population of our retrospective cohort study was derived from the Birth Registry System in Southern Zhejiang province, East China. We were given the medical records and home and work addresses of mother and term singleton live birth neonates who were delivered in hospital or childcare institutions from January 1, 2020 to December 31, 2022. Datasets stemmed from mothers’ medical records included maternal age, height, weight, gravidity, parity, gestational age (GA) at birth, medical conditions (e.g., cervical cerclage surgery (CCS), hypertension, preeclampsia, polyhydramnios, diabetes, GDM, intrahepatic cholestasis syndrome (ICS), hyperlipidemia, polycystic ovary syndrome (PCOS), PROM, uterine fibroids, leukocyte count, neutrophil granulocyte count, C reactive protein (CRP) level, and erythrocyte count), microbial infections (e.g., gonococcus, Group B streptococcus (GBS), *Mycoplasma hominis*, and *Ureaplasma urealyticum*), medical treatment (e.g., antibiotics, dexamethasone (DXM), magnesium sulphate, ritodrine, nifedipine, m-phenyltriphenylene, indomethacin suppository, dydrogesterone, and progesterone), pregnancy mode (e.g., natural, *in vitro* fertilization (IVF), and artificial insemination), delivery mode (e.g., natural, cesarean, and vagina assisted), intraoperative bleeding, personal life style (e.g., smoking and alcohol consumption), and treatment time (e.g., from hospital admission to delivery and from delivery to discharge). Neonate-related datasets include date of birth, birthweight, infant sex, fetal intrauterine distress, asphyxia, and APGAR scores (one minute and five minutes after delivery).

GA was defined as the period from ultrasound examination or last menstrual period reported by mothers to delivery. This study was approved by the ethics committee of School of Medicine, Zhejiang University (Trial No. 2024−1181). All study procedures were conducted in accordance with the guidelines of the Declaration of Helsinki. All participants enrolled in the study were completely anonymous and no individually identifiable information was given.

### 2.2 Exposure assessment

Participants’ home and work addresses were precise to the level of a specific residential compound, apartment complex, gated community, or block, where the maximum distance between any point within that block and the actual address was typically less than 500 meters. The atmospheric monitoring stations can continuously monitor hourly concentration of SO_2_. At least one atmospheric monitoring station can be found within a three-kilometer radius of the residential and work addresses of the participants we selected. All monitoring equipment was provided by Focused Photonics (Hangzhou), Inc. SO_2_ was measured by fluorescence using AQMS-500 SO_2_ Analyzer.

Maternal SO_2_ exposure levels were estimated using a time-activity weighted model. Due to strict government’s COVID-19 lockdown from early February to June 2020, we perceived that during this period, pregnant women had completely stayed at home, thus we used the data from atmospheric monitoring station closest to participants’ home addresses to assess exposure levels. During periods other than COVID-19 lockdown, exposure levels were calculated from two parts: weekdays and weekend. On weekdays, the exposure level from 8_A.M._ to 5_P.M._ was calculated based on data from the nearest atmospheric monitoring station to the pregnant women’s workplaces; the exposure level from 6 P_. M._ to 7 A_. M._ the next morning was assessed based on data from the nearest atmospheric monitoring station to the pregnant women’s home addresses. In weekend, the exposure level was calculated based on the data from the closed station to participants’ home addresses. Meteorological conditions were not considered in this study (https://data.cma.cn/).

### 2.3 Statistical analysis

We used Statistical Product and Service Solutions (SPSS) software package (https://spssau.com/) and the R Foundation for Statistical Computing (version 4.5.1) to conduct statistical analyses. The *p*-value, regression coefficient (β-value), and 95% confidence interval (CI) of β-value were used to assess the association between two datasets. Odds ratio (OR) and its 95% CI were used to assess the association. Cumulative effects of maternal SO_2_ exposure in three trimesters (first: from 1^st^ to 12^th^ weeks; second: from 13^th^ to 27^th^ weeks; third: 28^th^ to 37^th^ weeks) and during the entire pregnancy were estimated.

### 2.4 Machine learning models

Three machine learning models, including Random Forest (RF), XGBoost (XGB), and Multi-layer Perceptron (MLP), were used to predict birthweight. The RF model was configured with 500 trees, a maximum depth of 15, minimum samples per split of 10, and minimum samples per leaf of 5. The number of features considered at each split was set to the square root of the total number of features, following standard practice. For XGB, learning rate was 0.05, number of estimators was 300, and maximum depth was 5. For MLP parameters, hidden layer sizes were (128, 64, 32), learning rate was 0.001, alpha (L2 regularization) was 0.001, and batch size was 64. **Supplementary Tables 1**, **2** define the variables and their coding (dichotomous or tripartite). The 90/10 train-test split (10-fold cross-validation) was used. The procedure was repeated ten times to ensure that every observation received a predicted value.

Model performance was evaluated by comparing observed infant birthweight and predicted birthweight generated from machine learning models. Predictive accuracy was quantified using mean absolute error (MAE) and root mean squared error (RMSE), both expressed in grams. Due to gender differences in the impact of air pollution exposure on health (27), we divided participants into subgroups by neonatal sex.

### 2.5 Sensitivity analyses

We conducted sensitivity analyses to show the robustness of the results. To assess the impact of cross-regional variation in air pollution on effect estimates, we demonstrated our analyses by excluding the districts with smallest and largest number of participants who lived there, and also analyzed separately for urban and rural areas.

## 3 Results

### 3.1 Descriptive clinical statistics

**Supplementary Tables 3**–**S6** report birthweight with sex-specific stratification—mean ± standard deviation (SD)—for male and female neonates, respectively, across variables. Male neonates consistently had higher mean birthweight than females across almost all categories, which was physiologically expected and supported the internal validity of the dataset. Larger birthweight reductions (more than 150 g difference) were seen with ritodrine, dydrogesterone, DXM, ureaplasma urealyticum infection, GBS infection, gonococcal infection, and smoking. Moderate reductions (between 100 and 150 g) were associated with preeclampsia, chronic hypertension, ICS, and other comorbidities. GDM, chronic maternal diabetes, and uterine fibroids were linked to slightly higher birthweight. Inconsistent differences were shown between PCOS and birthweight.

After removing all potential influencing factors (including maternal illnesses, medical treatments, infections, smoking, and alcohol consumption), we recorded 21, 777 term singleton births in Southern Zhejiang province during January 2020 to December 2022 (**Figure 1**). The average maternal age was 30.67 ± 4.08 years old (**Supplementary Figure 1**). The maximum and minimum ages of the parturient women were 50 and 18 years old, respectively. Maternal height ranged from 140.5 to 180.0 cm, with an average of 160.75 ± 4.75 cm (**Supplementary Figure 2**). Average maternal weight was 68.06 ± 8.58 kg, ranging from 40.5 to 115.0 kg (**Supplementary Figure 3**). Mean maternal BMI was 26.33 ± 3.07, with maximum and minimum of 46.57 and 15.94, respectively (**Supplementary Figure 4**). Mean gravidity was 2.01 ± 1.11, ranging from 1 to 9 (**Supplementary Figure 5**). Mean parity was 1.47 ± 0.55, ranging from 1 to 5 (**Supplementary Figure 6**). Mean previous abortion times was 0.53 ± 0.85, ranging from 0 to 7 (**Supplementary Figure 7**). Mean GA was 38.92 ± 1.23, ranging from 37 to 41 (**Supplementary Figure 8**). The distributions of maternal characteristics and neonatal birthweight are given in **Figure 2**. Linear association of birthweight with maternal age, height, weight, BMI, gravidity, parity, number of previous abortions, GA, white blood cell count, neutrophil granulocytes, and CRP are shown in **Supplementary Tables 7**, **S8**. All variables exhibited statistically significant associations (p < 0.01) for both sexes. Sexual dimorphism was not observed.

**Figure 1:**
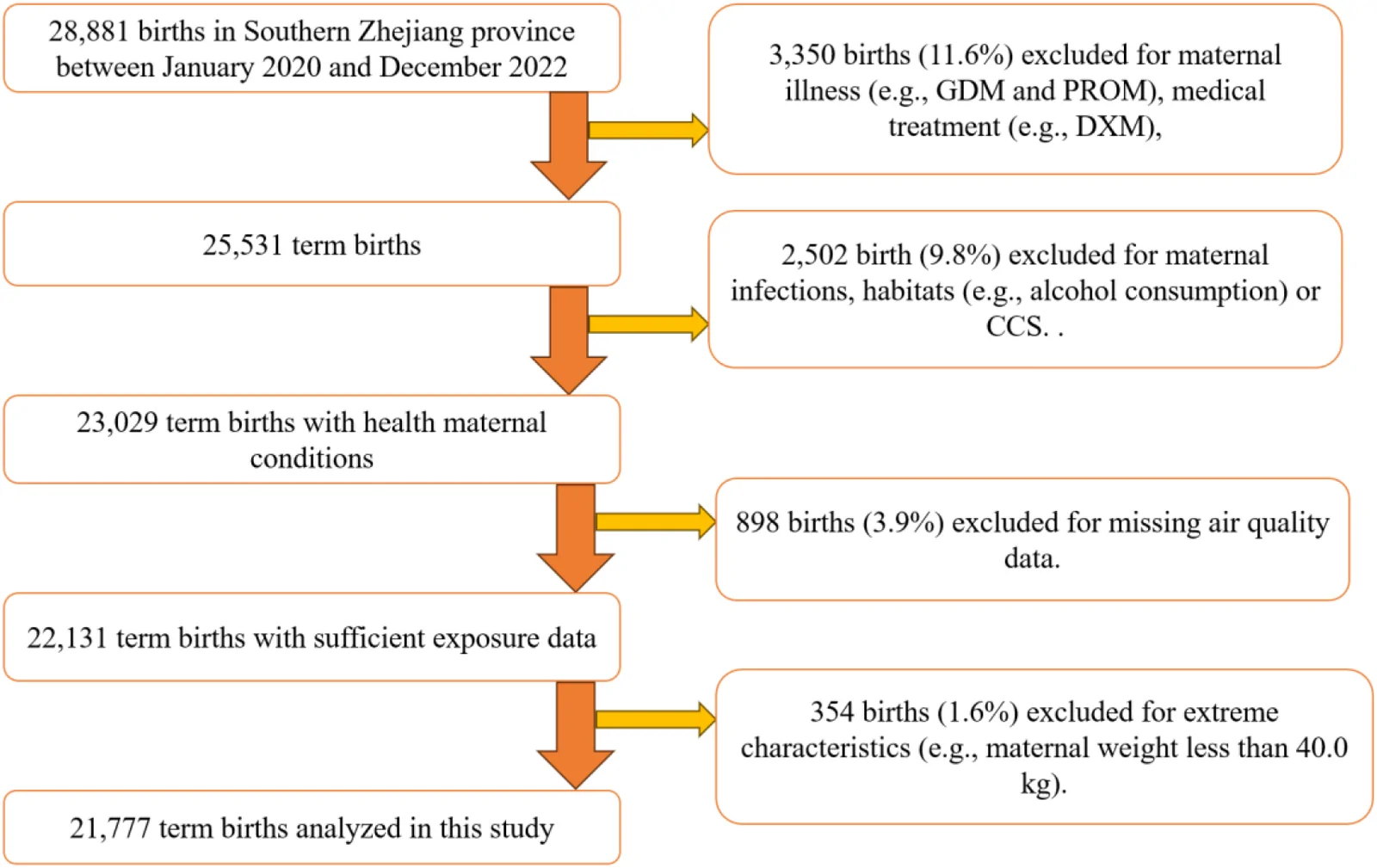
Selection of participants.

**Figure 2:**
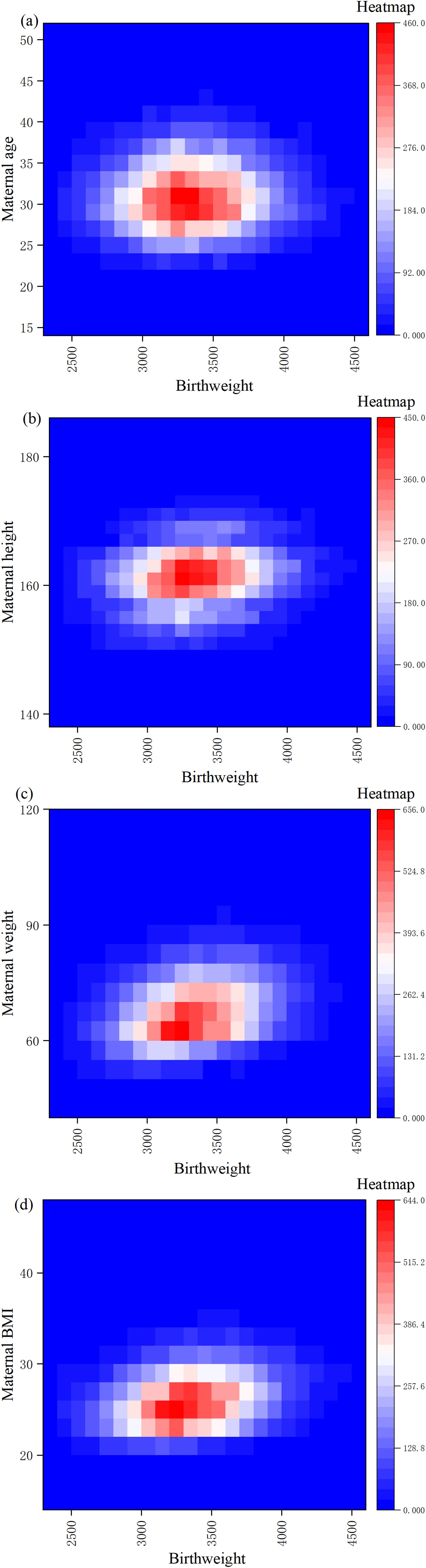
Distributions of maternal characteristics and neonatal birthweight.

### 3.2 Impact of COVID-19 lockdown on atmospheric SO_2_ levels

The COVID-19 pandemic and lockdown acted as a large-scale natural experiment for atmospheric SO_2_ levels, which was a unique opportunity to observe a sharp, exogenous reduction in ambient SO_2_ levels that allowed scientists to examine the exposure-response relationship under a wider range of exposure conditions than would typically be observed in a stable regulatory environment. We took SO_2_ level in the center monitoring station of the downtown for an example, as shown in **Supplementary Figure 9**. There was an immediate and sharp decline in early 2020, followed by a gradual, non-linear recovery through 2022. The COVID 19 pandemic induced a structural downward shift in SO_2_ levels, although this effect diminished over time as economic and industrial activities gradually resumed. However, the pre-pandemic concentration baseline was not reattained, highlighting a long-term downward adjustment in the city’s atmospheric SO_2_ burden, interspersed with strong seasonal and meteorological noise.

The annual average concentration during the pre-pandemic baseline in 2019 at the center monitoring station was about 7.86 µg/m³. The annual average dropped sharply to around 5.66 µg/m³ in 2020, the first year of the COVID-19 lockdown, representing a significant decrease of 28% compared to 2019. The annual average rebounded slightly to 6.25 µg/m³ in 2021, but remained approximately 20% lower than the 2019 level. The annual average continued to rise to 6.81 µg/m³ in 2022, which was approximately 13% lower than 2019, but notably higher than the 2020 and 2021 levels.

The strictest lockdowns in China occurred from late January to March 2020. A month-by-month comparison between 2019 and 2020 reveals a noticeable drop. The monthly mean atmospheric SO_2_ concentrations in January, February, March, and April 2020 decreased by 44.7%, 31.7%, 38.6%, and 37.3%, respectively, compared with the corresponding months in 2019. The most severe reduction occurred in January 2020, which coincided with the Lunar New Year holiday and the nationwide lockdown. The steep decline in February and March further confirms that reduced traffic, industrial shutdowns, and suspended construction activities directly curbed fossil fuel combustion emissions.

Despite the concentration shifts, the seasonal cycle remained robust across all four years. High concentrations occurred in winter due to increased coal combustion for heating, stagnant atmospheric conditions, and weaker vertical diffusion. Low concentrations occurred in summer, with July 2020 hitting the absolute record low of 2.91 µg/m^3^, which was likely due to a combination of lockdown aftereffects and favorable meteorological dispersion. The seasonal cycle indicated that while anthropogenic emissions dropped, meteorological factors (temperature, wind speed, precipitation) continued to dictate the monthly fluctuations.

A particularly noteworthy pattern emerged in April and May 2022. While 2020 and 2021 saw spring SO_2_ levels below 6 µg/m^3^, 2022 experienced a sudden spike (7.83 µg/m^3^ in April, the highest April value in the entire 4-year record). This contradicted the general “low pandemic level” trend. This anomaly can likely be attributed to: (1) the severe disruption to logistics and port operations in the neighboring Yangtze River Delta might have shifted local industrial operations or increased reliance on local auxiliary power generation; (2) unfavorable weather conditions (e.g., lower rainfall, weaker winds) in spring 2022 may have trapped pollutants locally, offsetting any emission reductions. The 2022 spring peak broke the monotonic decline, indicating that local meteorological conditions and specific regional logistic disruptions can sometimes outweigh the macro-pandemic emission-reduction effect.

### 3.3 IQR

**Supplementary Table 9** shows the 25^th^, 50^th^ (median), 75^th^ percentiles, and IQR. **Table 1** shows birthweight under different prenatal SO_2_ exposure levels. The average SO_2_ exposure during pregnancy was remarkably similar across both sexes and all gestational periods, with mean values ranging from 7.36 to 7.85 μg/m³. The IQR was consistently around 4.3–4.7 μg/m³, indicating a moderate spread in exposure levels among the study population. The exposure ranges (from minimum ~2.4 to maximum ~15.9 μg/m³) suggested a wide variability in air quality across different maternal residences or time points. Female fetuses were exposed to marginally higher average SO_2_ levels compared to males across all periods. However, these differences were extremely small and unlikely to be clinically or statistically significant. For both sexes, the third trimester consistently showed the lowest mean SO_2_ levels, while the first trimester showed the highest. As expected biologically, male neonates consistently had higher birthweights than female neonates for all exposure categories. This sex dimorphism was clearly preserved.

**Table 1:** Association between maternal SO_2_ exposure and birthweight.

Y	X (Maternal SO_2_ exposure)	Linear equation	95% CI	R^2^	*p*-value
Male					
Birthweight	First trimester	Y = -2.5671X + 3406.1	-4.944 ~ -0.191	0.0004	0.034
Birthweight	Second trimester	Y = -0.9364X + 3393.3	-3.312 ~ 1.439	5×10^-5^	0.044
Birthweight	Third trimester	Y = -2.4365X + 3399.6	-5.666 ~ -0.793	0.0004	0.009
Birthweight	Entire pregnancy	Y = -2.2547X + 3403.3	-5.889 ~ 1.380	0.0001	0.024
Female					
Birthweight	First trimester	Y = -2.755X + 3307.4	-5.135 ~ -0.375	0.0005	0.023
Birthweight	Second trimester	Y = -2.9194X + 3307.9	-5.314 ~ -0.525	0.0006	0.017
Birthweight	Third trimester	Y = -5.6073X + 3327.0	-8.042 ~ -3.172	0.002	<0.001
Birthweight	Entire pregnancy	Y = -4.0791X + 3316.8	-7.709 ~ -0.449	0.0005	0.028

**Supplementary Table 10** shows statistical association between maternal SO_2_ exposure and birthweight. The R² values across all trimester periods were low, indicating that maternal SO_2_ exposure solo explained less than 0.2% of the variance in birthweight, despite statistical significance in some associations. For male neonates, significant negative association was shown during the first trimester: a 1-unit (μg/m^3^) increase in SO_2_ was linked to a ~2.57 g decrease in birthweight. Association during the second trimester was not statistically significant. Significant negative association was revealed during the third trimester. The 95% CIs were wide. Early and late pregnancy exposure showed statistically significant negative effects, but the mid-gestation period showed no effect. The overall pregnancy exposure was not significant.

For female neonates, significant negative association was found, similar in magnitude to males. Highly significant negative associationwas shown during the third trimester, which was the largest negative effect size observed across all groups, indicating that the third trimester demonstrated the most substantial detrimental impact. Significant negative association was found during the entire pregnancy.

For susceptibility in sex-specific comparisons, females appeared more sensitive to SO_2_ exposure during the third trimester and across the entire pregnancy. For window-specific effects, the effect was limited to the first and third trimesters for male neonates; the effect spanned the first, third, and entire pregnancy for female neonates. While several p-values were below 0.05, the 95% CI for the slopes were generally wide.

### 3.4 SGA and LGA

**Supplementary Table 11** stratifies the association between maternal SO_2_ exposure and birthweight by neonatal sex across three weight-for-gestational-age categories, including SGA, appropriate for GA (AGA), and LGA. Exposure levels were presented for specific pregnancy windows, alongside birthweight means for each category. The birthweights of male neonates were heavier than females within every corresponding category.

In both sexes, a consistent dose-response pattern was observed across all trimesters: SGA mothers had the highest SO_2_ exposure; LGA mothers the lowest SO_2_; AGA mothers consistently fell in the middle. This gradient (SGA > AGA > LGA) supported the hypothesis that higher ambient SO_2_ exposure during pregnancy was associated with restricted fetal growth, while lower exposure trends toward larger birth size. The first trimester showed the highest exposure levels, making it a priority window for pollution intervention strategies.

### 3.5 Odds ratio

OR can be used to depict associations between prenatal exposure to air pollution and term LBW (28). Here we used OR to evaluate the link between prenatal SO_2_ exposure and birthweight. **Figure 3** presents β-coefficients and ORs with 95% CIs for each trimester and the entire pregnancy. An OR > 1 indicates an increased risk of the outcome per unit increase in SO_2_ exposure, while an OR < 1 indicates a decreased risk. Statistical significance is indicated when the 95% CI does not cross 1.00.

**Figure 3:**
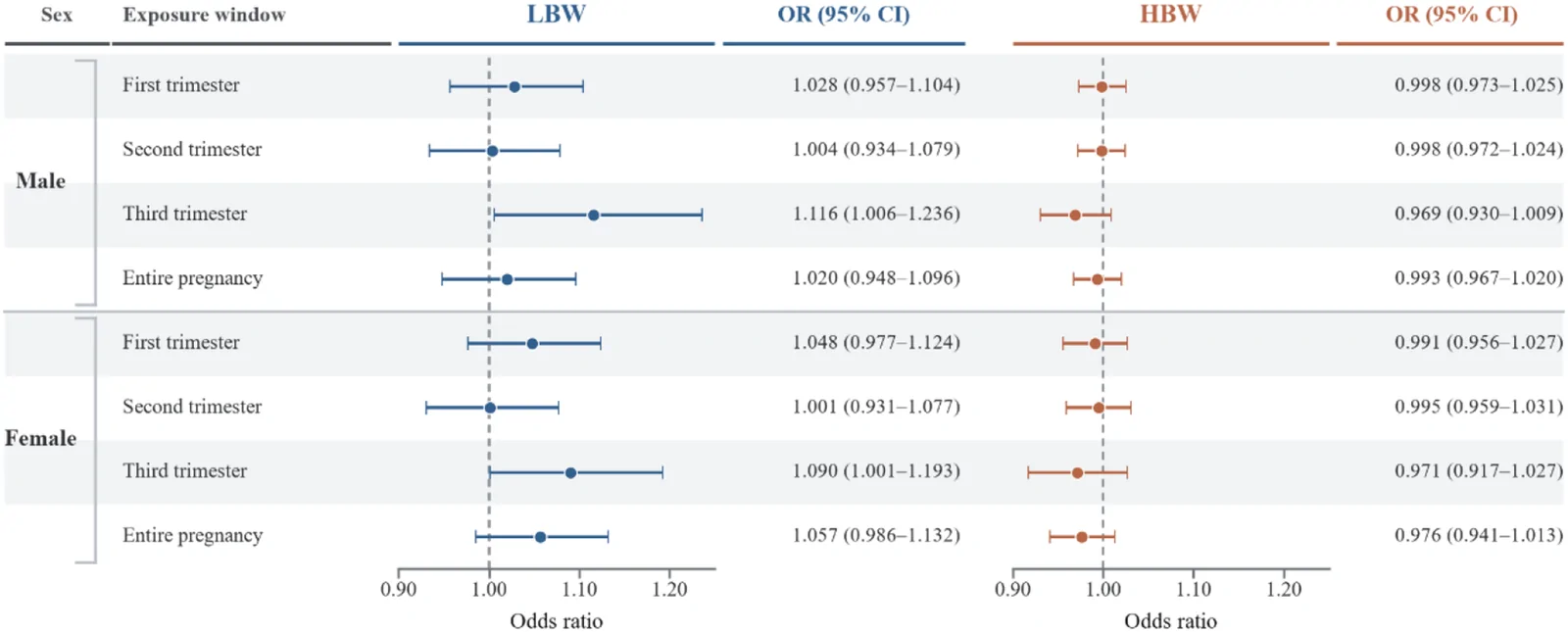
ORs of LBW and HBW infants to maternal SO_2_ exposure.

For male neonates, maternal SO_2_ exposure in the third trimester showed a statistically significant positive association with LBW, which suggested that each unit (1 μg/m^3^) increase in SO_2_ exposure throughout gestation was associated with an 11.6% increase in the odds of LBW. Although the ORs for the first and second trimesters and entire pregnancy were all greater than 1, none of them were statistically significant, because all 95% CIs crossed 1.0. For female neonates, the OR of LBW for the third trimester was marginally statistically significant. Similar to male infants, the ORs for the first and second trimester and entire pregnancy were all above 1.0 but lacked statistical significance, as all 95% CIs crossed 1.0. Prenatal SO_2_ exposure in late pregnancy was consistently associated with increased LBW risk in both sexes, with a slightly stronger and more robust effect observed in male infants.

For males, all ORs of HBW were below 1.0 during all exposure windows, suggesting a potential inverse relationship that SO_2_ exposure might slightly reduce the odds of HBW. However, none of these associations were statistically significant, as all 95% CIs included 1.0. For female neonates, similarly, all ORs were less than 1.0, indicating a protective trend. While the directional trends suggested that SO_2_ exposure may mildly suppress fetal HBW in both sexes, the effect was not statistically significant in any subgroup or time window, showing that the inverse association was not statistically significant at the conventional α=0.05 level.

### 3.6 Feature importance

As **Supplementary Tables 7**, **S8** shows, the R² values were universally low, indicating that none of the individual or laboratory factors, when used alone in the linear regression models, explained more than 8.2% of the variance in birthweight. The combined predictive utility of these univariate models was practically unknown, despite statistical significance driven by a likely large sample size. We used machine learning models to test the birthweight prediction accuracy.

Feature Importance (FI) can measure the relative contribution of input parameter to the machine learning model’s prediction outcomes. **Table 2** presents the normalized percentage contributions (summing to 100% for each model) of 20 clinical, demographic, and environmental predictors, which were used to predict neonatal birthweight. Both models (RF and XGB) agreed on the overall hierarchy of key drivers, but they differed significantly in how aggressively they weight certain top-tier features. Both algorithms consistently identified GA, maternal weight, and neonatal gender as the three most influential factors. GA was the undisputed strongest predictor in both models; XGB showed greater FI than RF did, suggesting that XGB more relied on GA as a primary splitting criterion, while RF distributed predictive power more evenly across features. Maternal weight ranked second in both models, reinforcing that maternal nutritional and metabolic status were critical biological determinants. Neonatal gender was the third most important feature, confirming known physiological differences in fetal growth trajectories.

**Table 2:** Feature importance.

Features	RF (100%)	XGB (100%)
Maternal age	5.5%	1.5%
Maternal height	4.6%	2.5%
Maternal weight	10.0%	15.7%
Maternal BMI	4.0%	2.8%
Gravidity	4.2%	3.1%
Parity	4.3%	7.2%
Number of previous abortions	1.7%	2.1%
GA	10.9%	22.1%
Pregnancy mode	4.0%	2.7%
Delivery mode	4.8%	6.0%
Body temperature during delivery	1.1%	2.9%
White blood cell count	3.8%	2.7%
Neutrophil granulocytes	6.2%	2.8%
CRP	3.0%	2.4%
Home location (urban or rural)	2.8%	2.1%
Neonatal gender	7.3%	10.3%
SO_2_ exposure in the first trimester	5.5%	2.5%
SO_2_ exposure in the second trimester	5.3%	2.7%
SO_2_ exposure in the third trimester	5.6%	2.8%
SO_2_ exposure during the entire pregnancy	5.4%	3.1%

For model discrepancies, XGB exhibited a more “spiked” FI distribution. The top three features (GA, maternal weight, and gender) collectively accounted for 48.1% of XGB’s total predictive power, whereas the three accounted for only 28.2% in RF, indicating that XGB prioritized a small set of powerful variables, whereas RF spread its importance across a wider array of traits to build robust ensembles.

RF assigned notably higher FI to neutrophil granulocytes and a slightly higher weight to white blood cell count, suggesting that RF was more sensitive to capturing subtle immune-inflammatory/immune signals during pregnancy, whereas XGB largely dismissed them as secondary noises. RF consistently valued maternal SO_2_ exposure across all trimesters at a moderate level. In stark contrast, XGB assigned consistently low importance to all SO_2_-related variables. This divergence implied that RF may be capturing non-linear interactions between air pollution and other clinical factors, while XGB likely overrode these effects with GA and maternal weight. Moreover, in the RF model, maternal SO_2_ exposure showed remarkably stable importance across all trimesters. This near-identical distribution suggested that, in RF’s view, the cumulative or average exposure during pregnancy matters equally across stages, rather than any specific “critical window” being exceptionally more sensitive. Several features (e.g., previous abortion times, CRP, home location, and pregnancy modes) were ranked low in both models, indicating limited individual contribution to birthweight prediction. These variables likely had indirect effects that were mediated through other factors (e.g., BMI or GA) rather than contributing direct independent predictive value.

From a clinical perspective, fetal maturity (e.g., GA), maternal metabolic reserves (e.g., maternal weight), and gender were the absolute cornerstones of determining factors of birthweight prediction. While XGB delivered a more parsimonious, high-leverage model heavily anchored on these variables, RF provided a more holistic view—capturing environmental (SO_2_) and hematological (neutrophils) contributions that XGB largely ignored. If interpretability and clinical consensus were the goal, RF’s balanced weighting may be more intuitive; if pure predictive performance was prioritized, XGB’s sharp focus on GA and maternal weight might yield higher efficiency, provided these key variables can be reliably measured.

### 3.7 Birthweight prediction

Despite the β-coefficients in linear regression models (**Figure 4**), machine learning models may better capture the non-linear association of birthweight with clinical and demographic predictors (**Figure 5**). Association between birthweight and GA in this analysis was similar to the previous study (29). We used RF, XGB, and MLP in a two-step approach to predict neonatal birthweight. In the first step, the input parameters consisted of the 16 standard clinical and demographic features in **Table 2**, excluding maternal SO_2_ exposure. In the second step, maternal SO_2_ exposure levels were also added as input parameters. By comparing the improvement in prediction performance between the two steps, we could quantitatively assess the potential impact of maternal SO_2_ exposure on birthweight.

**Figure 4:**
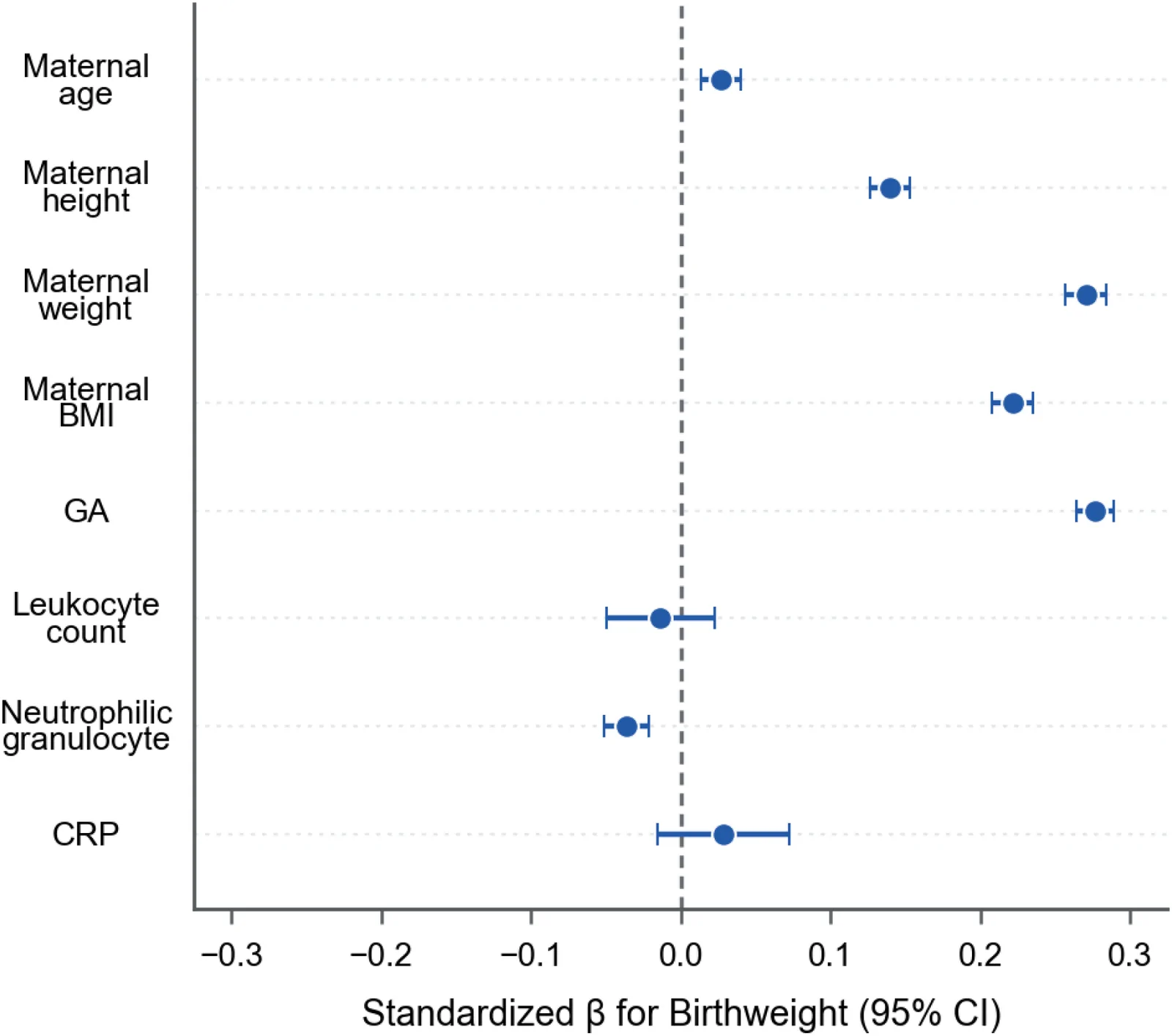
β-coefficients between birthweight and maternal traits.

**Figure 5:**
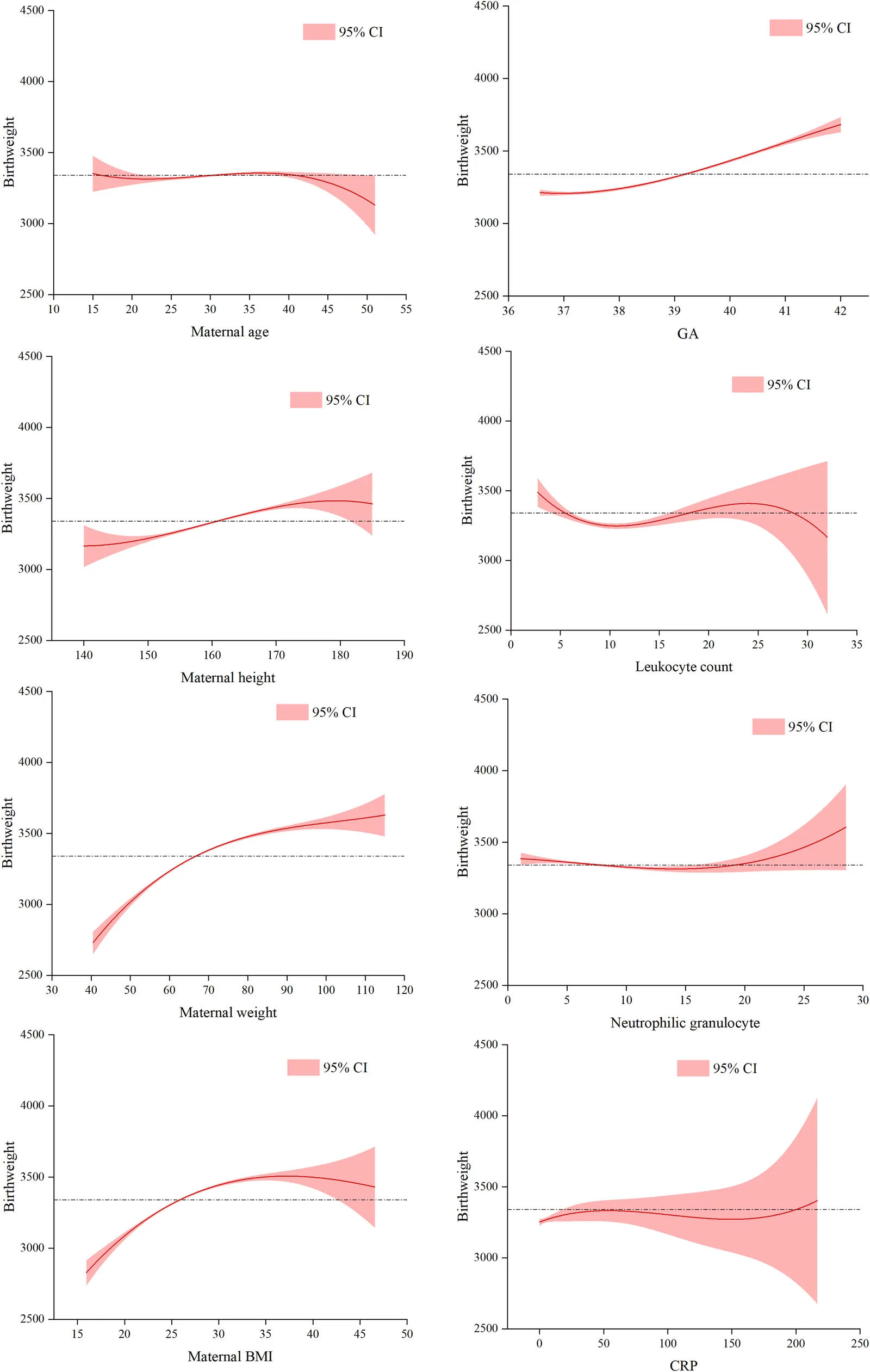
Non-linear association of birthweight with clinical and demographic predictors.

**Supplementary Table 12** shows the birthweight prediction results. For all three machine learning models, when maternal SO_2_ exposure levels were added as input parameters, consistent reductions in prediction error were shown, indicating that SO_2_ exposure was a useful informative feature. The improvement was most noticeable in neonates with extreme birthweights (e.g., <2500g or >4000g), where SO_2_ exposure likely acted as a risk modifier. XGB prediction was more accurate than the RF model. MLP showed the smallest relative improvement, suggesting that while adding maternal SO_2_ exposure enhanced prediction accuracy, MLP may need more architectural tuning (e.g., layers, regularization) to fully exploit the additional features. Paired t-tests (comparing absolute errors for each record) showed p less than 0.001 for all three models, indicating statistically significant improvements. The improvements were consistent across all GA groups, but more pronounced when GA was 41 weeks, where SO_2_ had a longer cumulative effect. Overall, the accuracy improvements in predicting birthweight were notable, especially for high-risk pregnancies such as LBW and HBW.

## 4 Discussion

This study provides novel evidence on the association between maternal SO_2_ exposure and birthweight by leveraging the unique quasi-experimental conditions created by the COVID-19 lockdown in East China. Our findings demonstrate three major factors: (1) confirmation of a significant negative association between SO_2_ exposure and birthweight, with sex-specific susceptibility windows; (2) quantification of increased LBW risk associated with SO_2_ exposure in the third trimester; and (3) demonstration that incorporating prenatal SO_2_ exposure into machine learning models improved birthweight prediction accuracy.

Sex-specific susceptibility and critical windows were identified. Our results revealed notable sexual dimorphism in response to maternal SO_2_ exposure. Female neonates exhibited stronger negative associations during the third trimester and across the entire pregnancy, suggesting greater vulnerability. This finding aligns with previous studies reporting sex-specific effects of air pollution on fetal growth (27), potentially due to differences in placental development, hormonal regulation, or oxidative stress responses between male and female fetuses. The third trimester emerged as the most sensitive period for both sexes, consistent with the rapid fetal fat accretion and weight gain occurring during this stage.

Clinical significance of effect sizes was meaningful. At the individual level, a reduction of several grams in birthweight was unlikely to be clinically detectable, measurable, and actionable. However, public health implication at large population level may be significant. Given the widespread exposure distribution across the entire Chinese population, even modest effect sizes can translate into meaningful shifts in the distribution of birthweight and an increased proportion of LBW infants. This was particularly relevant for pregnancies already at marginal nutritional or metabolic status.

The ORs for LBW indicated statistically significant risk elevations, particularly for pregnancies already at marginal nutritional or metabolic status. The consistent dose-response gradient observed across SGA, AGA, and LGA categories further supported the biological plausibility of SO_2_-induced growth restriction.

Mechanistic insights were provided. The observed epidemiological associations may involve oxidative stress, systemic inflammation, or placental dysfunction induced by SO_2_ and its secondary products (sulfates), although direct biomarker evidence was absent. The elevated neutrophil counts and their moderate FI in the RF model indirectly supported an inflammatory pathway. Notably, the CRP and white blood cell counts showed weaker contributions, suggesting that SO_2_ may exert its effects through pathways beyond systemic inflammation, potentially involving epigenetic modifications or endocrine disruption.

Our findings addressed the previously inconsistent literature on SO_2_ and birthweight (10, 12). The use of high-resolution exposure assessment and the natural experiment design reduced confounding by unmeasured behavioral or policy factors that frequently complicated observational studies. The effect sizes were comparable to those reported for PM_2.5_ (21) and O_3_ (17), positioning SO_2_ as a non-negligible environmental contributor to adverse birth outcomes.

The consistent improvement in prediction accuracy across all three machine learning models when adding SO_2_ exposure as input confirmed its informative value beyond traditional clinical factors. The divergent FI patterns between RF and XGB highlighted different modeling philosophies: RF’s balanced weighting may better capture non-linear interactions between environmental and clinical factors, while XGB’s sharp focus on GA and maternal weight yielded higher predictive efficiency. This complementarity suggested that ensemble approaches combining both algorithms could optimize clinical decision support. The statistically significant but modest RMSE reductions indicated that while SO_2_ was not a dominant predictor, its inclusion provided incremental value, especially for extreme birthweight cases where environmental exposure may act as more impactful risk modifiers.

We listed several strengths and limitations. Major strengths included the large, population-based samples with high-quality clinical data, precise geocoded exposure assessment accounting for time-activity patterns, the quasi-experimental COVID-19 lockdown design, and rigorous machine learning evaluation with cross-validation. However, several limitations must be acknowledged. First, weather plays an important role in modifying child health outcomes (30), but we did not account for meteorological conditions (e.g., temperature, wind speed, and precipitation) that may modify SO_2_ dispersion and individual exposure levels. We did not obtain official meteorological information because of the need for data confidentiality. Second, our monitoring-based estimates did not account for indoor sources (e.g., coal or biomass combustion for heating or cooking), which might disproportionately affect rural participants and could introduce exposure misclassification. Third, the study was conducted in a single Chinese province with relatively low atmospheric SO_2_ levels, limiting generalizability to highly polluted regions. Fourth, we assumed complete home confinement during lockdown and quarantine periods, which may not reflect all real individual behaviors. Fifth, while our birth registry data did not contain individual level information on education, income, occupation, detailed dietary intake, and maternal weight gain during pregnancy, we fully acknowledge that these socioeconomic and nutritional factors could influence both exposure patterns and birth outcomes, particularly across rural settings. Sixth, the “natural experiment” primarily applied to ambient SO_2_ exposure levels, rather than to individual level birth outcomes. Our analysis of birth outcomes was cross sectional within the post-lockdown period, and that we leveraged the variation in SO_2_ exposure during this period—induced by the lockdown and subsequent recovery—to assess exposure-response associations. Lockdown served as providing a quasi-experimental opportunity to observe exposure variation rather than a pre/post natural experiment for birth outcomes. Pre-pandemic birth cohorts were not included to directly compare birth outcomes before and after large scale emission changes. Seventh, our hematological data (e.g., neutrophil counts, CRP, and white blood cell counts) provided only indirect and limited support for an inflammatory pathway, and that these findings were hypothesis-generating rather than confirmatory. Finally, the retrospective design precluded causal inference despite the natural experiment framework.

Our study gave public health implications. The findings underscored the need for continued air quality management even during economic recovery periods, as evidenced by the incomplete return to pre-pandemic SO_2_ levels by 2022. Clinically, obstetricians should consider maternal residential air pollution levels when assessing LBW risk, particularly in the third trimester. The successful integration of environmental data into machine learning prediction models offered a practical tool for early identification of high-risk pregnancies, enabling targeted nutritional support, enhanced monitoring, and timely interventions. We argue that SO_2_ was a non-negligible rather than a dominant risk factor, and that its public health importance lay in its population level impact due to widespread exposure, rather than its individual predictive power.

As for future directions, longitudinal studies with individual-level personal exposure monitoring (e.g., portable sensors) are needed to validate our findings and reduce exposure misclassification. Mechanistic investigations exploring placental epigenetic changes, oxidative stress biomarkers, and endocrine disruption pathways would elucidate biological mechanisms. The cost-effectiveness of incorporating environmental data into routine antenatal care should be evaluated in implementation research. Finally, interventional studies targeting air pollution reduction (e.g., community-level policies, personal protective measures) could directly test the causal relationship and inform public health policy. Directly measuring placental oxidative stress markers, inflammatory cytokines, epigenetic modifications, and hormonal profiles to elucidate the underlying biological mechanisms linking SO_2_ exposure to restricted fetal growth is needed.

## 5 Conclusion

This study explored the association between maternal SO_2_ exposure and birthweight reduction during the COVID-19 lockdown and quarantine period, using a large retrospective cohort and machine learning prediction models to assess the incremental contribution of air pollution to birthweight prediction. Our analysis examined the exposure-response relationship during the 2020–2022 period, leveraging the lockdown-induced variation in air quality as a natural experiment for exposure, not for birth outcomes. The COVID-19 lockdown provided a unique opportunity to observe these associations under drastically altered emission patterns, strengthening the evidence base for SO_2_ as a non-negligible risk factor. Maternal SO_2_ exposure during pregnancy, particularly in the third trimester, was significantly associated with reduced birthweight and increased odds of LBW, with female and male fetuses exhibiting greater susceptibility, respectively. The integration of SO_2_ exposure data into machine learning models improved birthweight prediction accuracy, supporting the clinical utility of environmental risk assessment in antenatal care. These findings highlight the dual importance of continued air quality regulation and enhanced clinical surveillance for pregnancies in polluted areas. To optimize maternal and neonatal health, public policies should prioritize sustained reduction in ambient SO_2_ levels, while healthcare systems should consider incorporating environmental exposure data into personalized risk stratification tools. Given the prediction results of machine learning models, we perceive that traditional linear regression models may overestimate the impact of air pollutants on adverse reproductive outcomes. Future research should focus on elucidating biological mechanisms and evaluating the effectiveness of targeted interventions in high-risk populations.

## Data Availability

The raw data supporting the conclusions of this article will be made available by the authors, without undue reservation.
