# Supplementary material for: Maternal SO2 exposure and birthweight reduction during COVID-19 lockdown and quarantine in East China
Source: Front Endocrinol (Lausanne). 2026 Sep 18;17:1954694. doi: 10.3389/fendo.2026.1954694 (PMC13630596; doi:10.3389/fendo.2026.1954694)
Supplement: Supplementary file 1 [file Supplementaryfile1.docx]

**Supplementary Materials for**

**Maternal SO_2_ exposure and birthweight reduction during COVID-19 lockdown and quarantine in East China**

The PDF file includes:

Tables S1 to S12

Figures S1 to S9

Table S1 | Dichotomous

| Variables | 0 | 1 |
| --- | --- | --- |
| Body temperature during delivery | Less than 37.5 °C | More than 37.5 °C |
| Smoking tobacco during pregnancy | No smoking | Smoking |
| Drinking alcohol during pregnancy | No drinking | Drinking |
| Polyhydramnios | No | Yes |
| Chronic maternal diabetes | No | Yes |
| GDM | No | Yes |
| Preeclampsia | No | Yes |
| ICS | No | Yes |
| Chronic hypertension | No | Yes |
| Hyperlipidemia | No | Yes |
| PCOS | No | Yes |
| Uterine fibroids | No | Yes |
| PROM | No | Yes |
| Group B streptococcus infection | No | Yes |
| Gonococcal infection | No | Yes |
| Mycoplasma hominis | No | Yes |
| Ureaplasma urealyticum | No | Yes |
| Antibiotic | Did not use during pregnancy | Used during pregnancy |
| DXM | Did not use during pregnancy | Used during pregnancy |
| Magnesium sulphate | Did not use during pregnancy | Used during pregnancy |
| Ritodrine | Did not use during pregnancy | Used during pregnancy |
| Nifedipine | Did not use during pregnancy | Used during pregnancy |
| m-phenyltriphenylene | Did not use during pregnancy | Used during pregnancy |
| Indomethacin suppository | Did not use during pregnancy | Used during pregnancy |
| Dydrogesterone | Did not use during pregnancy | Used during pregnancy |
| Progesterone | Did not use during pregnancy | Used during pregnancy |
| Fetal intrauterine distress | No | Yes |
| Address | Rural | Urban |

Table S2 | Tripartite

| Variables | 0 | 1 | 2 |
| --- | --- | --- | --- |
| Pregnancy mode | Natural | IVF | Artificial insemination |
| Delivery mode | Natural | Cesarean | Assisted |
| Anemia (erythrocyte concentration) | Less than 60 | Between 60 and 110 | More than 110 |

Table S3 | Birthweight discrepancy of male neonates for dichotomous parameters

| Variables | 0 | 1 |
| --- | --- | --- |
| Body temperature during delivery | 3384.7 ± 386.1 | 3400.9 ± 412.5 |
| Smoking tobacco during pregnancy | - | - |
| Drinking alcohol during pregnancy | 3387.4 ± 388.6 | 3282.5 ± 386.1 |
| CCS | 3387.5 ± 388.3 | 3129.4 ± 385.9 |
| Polyhydramnios | 3386.2 ± 388.7 | 3351.4 ± 363.0 |
| Chronic maternal diabetes | 3377.9 ± 383.9 | 3423.8 ± 407.6 |
| GDM | 3382.2 ± 387.2 | 3419.7 ± 399.5 |
| Preeclampsia | 3388.0 ± 387.8 | 3200.3 ± 431.7 |
| ICS | 3387.2 ± 388.4 | 3327.6 ± 405.2 |
| Chronic hypertension | 3386.6 ± 388.5 | 3185.2 ± 440.5 |
| Hyperlipidemia | 3386.4 ± 389.1 | 3367.8 ± 336.9 |
| Anemia | 3386.6 ± 388.6 | 3333.5 ± 404.9 |
| PCOS | 3386.3 ± 388.6 | 3363.2 ± 423.2 |
| Uterine fibroids | 3386.0 ± 388.6 | 3430.4 ± 395.8 |
| PROM | 3398.3 ± 397.7 | 3383.8 ± 386.8 |
| Other comorbidities | 3387.6 ± 388.2 | 3296.0 ± 409.3 |
| Group B streptococcus infection | 3386.4 ± 388.6 | 3011.3 ± 469.8 |
| Gonococcal infection | 3387.1 ± 388.1 | 3207.7 ± 456.3 |
| Ureaplasma urealyticum | 3386.4 ± 388.6 | 2871.3 ± 343.4 |
| Antibiotic | 3383.2 ± 359.1 | 3386.7 ± 393.4 |
| DXM | 3389.9 ± 386.3 | 2994.3 ± 444.7 |
| Magnesium sulphate | 3396.7 ± 385.8 | 3319.4 ± 400.5 |
| Ritodrine | 3388.7 ± 386.6 | 2790.2 ± 415.2 |
| Nifedipine | 3389.6 ± 385.8 | 3292.2 ± 451.4 |
| m-phenyltriphenylene | - | - |
| Indomethacin suppository | - | - |
| Dydrogesterone | 3386.8 ± 388.2 | 2888.6 ± 436.3 |
| Progesterone | - | - |
| Fetal intrauterine distress | 3386.8 ± 388.6 | 3360.5 ± 392.6 |
| Home address | 3381.1 ± 394.1 | 3389.1 ± 385.6 |
| Work address | 3380.5 ± 384.2 | 3393.6 ± 394.2 |

Table S4 | Birthweight discrepancy of male neonates for tripartite parameters

| Variables | 0 | 1 | 2 |
| --- | --- | --- | --- |
| Pregnancy mode | 3385.8 ± 388.0 | 3426.1 ± 425.4 | 3283.6 ± 360.9 |
| Delivery mode | 3377.3 ± 371.4 | 3401.7 ± 415.6 | 3316.5 ± 302.5 |

Table S5 | Birthweight discrepancy of female neonates for dichotomous parameters

| Variables | 0 | 1 |
| --- | --- | --- |
| Body temperature during delivery | 3279.7 ± 368.9 | 3344.8 ± 394.4 |
| Smoking tobacco during pregnancy | 3285.6 ± 371.8 | 3150.0 ± 70.0 |
| Drinking alcohol during pregnancy | 3286.6 ± 371.4 | 3203.8 ± 385.9 |
| CCS | 3286.2 ± 371.5 | 3165.5 ± 392.4 |
| Polyhydramnios | 3285.6 ± 371.7 | 3183.3 ± 511.4 |
| Chronic maternal diabetes | 3280.8 ± 368.4 | 3307.7 ± 385.6 |
| GDM | 3282.7 ± 368.5 | 3309.8 ± 371.7 |
| Preeclampsia | 3286.5 ± 370.8 | 3188.2 ± 451.3 |
| ICS | 3287.0 ± 371.5 | 3185.8 ± 370.5 |
| Chronic hypertension | 3286.1 ± 371.6 | 3060.0 ± 331.6 |
| Hyperlipidemia | 3285.5 ± 371.5 | 3294.6 ± 397.1 |
| Anemia | 3285.5 ± 370.8 | 3295.9 ± 374.2 |
| PCOS | 3285.2 ± 371.4 | 3455.7 ± 461.5 |
| Uterine fibroids | 3285.7 ± 371.5 | 3260.5 ± 403.1 |
| PROM | 3295.3 ± 366.0 | 3283.6 ± 372.8 |
| Other comorbidities | 3286.9 ± 370.9 | 3195.7 ± 413.0 |
| Group B streptococcus infection | 3285.8 ± 371.6 | 2918.0 ± 400.2 |
| Gonococcal infection | 3313.3 ± 398.7 | 3285.5 ± 371.6 |
| Ureaplasma urealyticum | 3285.7 ± 371.6 | 2625.0 ± 125.0 |
| Antibiotic | 3272.9 ± 339.1 | 3287.8 ± 377.1 |
| DXM | 3287.7 ± 370.1 | 2952.4 ± 468.3 |
| Magnesium sulphate | 3293.3 ± 370.3 | 3232.2 ± 377.4 |
| Ritodrine | 3287.1 ± 370.1 | 2722.3 ± 511.7 |
| Nifedipine | 3283.9 ± 369.3 | 3217.0 ± 431.1 |
| m-phenyltriphenylene | - | - |
| Indomethacin suppository | 3285.7 ± 371.8 | 3175.0 ± 197.4 |
| Dydrogesterone | 3285.9 ± 371.0 | 2584.0 ± 888.9 |
| Progesterone | 3285.9 ± 371.1 | 3040.0 ± 217.7 |
| Fetal intrauterine distress | 3286.6 ± 370.6 | 3253.8 ± 386.0 |
| Home address | 3281.6 ± 371.7 | 3289.0 ± 370.5 |
| Work address | 3285.4 ± 370.3 | 3286.0 ± 371.5 |

Table S6 | Birthweight discrepancy of female neonates for tripartite parameters

| Variables | 0 | 1 | 2 |
| --- | --- | --- | --- |
| Pregnancy mode | 3286.4 ± 371.1 | 3261.5 ± 372.5 | 3245.9 ± 335.4 |
| Delivery mode | 3280.5 ± 356.2 | 3296.4 ± 394.9 | 3131.1 ± 397.3 |

Table S7 | Linear equation for birthweight of male neonates

| Y | X | Linear equation | R^2^ |
| --- | --- | --- | --- |
| Maternal age | Birthweight | Y = 0.0003 X + 29.528 | 0.0011 |
| Maternal height | Birthweight | Y = 0.0017 X + 154.91 | 0.0197 |
| Maternal weight | Birthweight | Y = 0.0051 X + 67.973 | 0.0625 |
| Maternal BMI | Birthweight | Y = 0.0016 X + 20.782 | 0.0434 |
| Gravidity | Birthweight | Y = 0.0002 X + 1.2227 | 0.0065 |
| Parity | Birthweight | Y = 0.0001 X + 1.1148 | 0.0056 |
| Number of previous abortions | Birthweight | Y = 0.0001 X + 0.1078 | 0.0033 |
| GA (weeks) | Birthweight | Y = 0.0009 X + 35.805 | 0.0821 |
| White blood cell count | Birthweight | Y = -9×10^-6^ X + 9.7379 | 10^-6^ |
| Neutrophil granulocytes | Birthweight | Y = -0.0003 X + 8.5116 | 0.0013 |
| CRP | Birthweight | Y = 0.0002 X + 5.7443 | 2×10^-5^ |

Table S8 | Linear equation for birthweight of female neonates

| Y | X | Linear equation | R^2^ |
| --- | --- | --- | --- |
| Maternal age | Birthweight | Y = 0.0002 X + 29.959 | 0.0004 |
| Maternal height | Birthweight | Y = 0.0018 X + 154.87 | 0.0198 |
| Maternal weight | Birthweight | Y = 0.0066 X + 46.217 | 0.0817 |
| Maternal BMI | Birthweight | Y = 0.002 X + 19.839 | 0.0568 |
| Gravidity | Birthweight | Y = 0.0002 X + 1.3449 | 0.0043 |
| Parity | Birthweight | Y = 0.0001 X + 1.1215 | 0.0048 |
| Number of previous abortions | Birthweight | Y = 10^-4^ X + 0.2234 | 0.0018 |
| GA (weeks) | Birthweight | Y = 0.001 X + 35.81 | 0.082 |
| White blood cell count | Birthweight | Y = -0.0002 X + 10.473 | 0.0007 |
| Neutrophil granulocytes | Birthweight | Y = -0.0003 X + 8.5463 | 0.0014 |
| CRP | Birthweight | Y = 0.0022 X – 0.7285 | 0.0035 |

Table S9. Maternal exposure to SO_2_ during pregnancy (μg/m^3^)

| Male | Minimum | 25th percentile | 50th percentile | 75th percentile | Maximum | IQR | Mean | SD |
| --- | --- | --- | --- | --- | --- | --- | --- | --- |
| First trimester | 2.86 | 5.25 | 7.30 | 9.90 | 15.43 | 4.65 | 7.76 | 3.00 |
| Second trimester | 2.97 | 4.83 | 7.01 | 9.46 | 15.24 | 4.63 | 7.52 | 2.98 |
| Third trimester | 2.41 | 4.86 | 6.96 | 9.36 | 15.95 | 4.60 | 7.39 | 2.92 |
| Entire pregnancy | 2.77 | 4.95 | 7.03 | 9.59 | 15.59 | 4.64 | 7.57 | 2.86 |
| Female |  |  |  |  |  |  |  |  |
| First trimester | 2.91 | 5.47 | 7.47 | 10.16 | 15.21 | 4.69 | 7.85 | 3.01 |
| Second trimester | 2.93 | 5.13 | 7.03 | 9.50 | 15.19 | 4.37 | 7.57 | 2.99 |
| Third trimester | 2.49 | 4.99 | 6.95 | 9.32 | 15.91 | 4.33 | 7.36 | 2.94 |
| Entire pregnancy | 2.57 | 5.12 | 6.93 | 9.43 | 15.60 | 4.31 | 7.61 | 2.97 |

Table S10 | Neonatal birthweight under different prenatal SO_2_ exposure levels (g)

| Male | <25^th^ percentile | 25^th-^50^th^ percentile | 50^th^ to 75^th^ percentile | >75^th^ percentile |
| --- | --- | --- | --- | --- |
| First trimester | 3395.7 ± 385.4 | 3384.8 ± 381.2 | 3389.6 ± 389.2 | 3374.7 ± 397.8 |
| Second trimester | 3392.7 ± 388.7 | 3390.8 ± 388.3 | 3377.4 ± 389.6 | 3384.0 ± 387.9 |
| Third trimester | 3395.7 ± 388.0 | 3389.0 ± 398.1 | 3388.6 ± 380.2 | 3371.6 ± 387.8 |
| Entire pregnancy | 3400.0 ± 387.3 | 3383.4 ± 387.0 | 3383.0 ± 390.3 | 3378.5 ± 389.8 |
| Female |  |  |  |  |
| First trimester | 3299.1 ± 361.9 | 3284.2 ± 375.9 | 3283.0 ± 373.8 | 3276.9 ± 372.0 |
| Second trimester | 3303.0 ± 366.4 | 3285.1 ± 366.2 | 3277.7 ± 373.9 | 3277.4 ± 376.9 |
| Third trimester | 3311.9 ± 364.0 | 3286.7 ± 375.0 | 3285.2 ± 373.0 | 3259.4 ± 370.2 |
| Entire pregnancy | 3299.0 ± 361.5 | 3295.3 ± 376.8 | 3277.2 ± 369.3 | 3271.7 ± 375.7 |

Table S11 | Association between maternal SO_2_ exposure and all neonates

|  | SGA |  | AGA |  | LGA |  |
| --- | --- | --- | --- | --- | --- | --- |
| Male | Birthweight | Exposure level | Birthweight | Exposure level | Birthweight | Exposure level |
| First trimester | 2716.2 ± 129.6 | 8.01 ± 3.03 | 3382.6 ± 258.3 | 7.73 ± 3.07 | 4085.0 ± 151.8 | 7.79 ± 2.98 |
| Second trimester |  | 7.54 ± 2.93 |  | 7.52 ± 3.01 |  | 7.49 ± 2.98 |
| Third trimester |  | 7.41 ± 2.96 |  | 7.39 ± 3.12 |  | 7.30 ± 2.86 |
| Entire pregnancy |  | 7.92 ± 2.94 |  | 7.67 ± 1.97 |  | 7.81 ± 1.91 |
|  |  |  |  |  |  |  |
| Female |  |  |  |  |  |  |
| First trimester | 2664.1 ± 112.5 | 8.05 ± 2.98 | 3277.6 ± 243.6 | 7.84 ± 3.01 | 3973.2 ± 172.1 | 7.80 ± 2.97 |
| Second trimester |  | 7.82 ± 2.98 |  | 7.55 ± 3.02 |  | 7.47 ± 2.92 |
| Third trimester |  | 7.67 ± 2.97 |  | 7.34 ± 3.06 |  | 7.22 ± 2.95 |
| Entire pregnancy |  | 7.99 ± 2.97 |  | 7.85 ± 1.98 |  | 7.81 ± 2.93 |

Table S12 | Birthweight prediction results

|  | RF | | XGB | | MLP | |
| --- | --- | --- | --- | --- | --- | --- |
|  | MAE | RMSE | MAE | RMSE | MAE | RMSE |
| Step 1 | 314.9 | 400.0 | 297.9 | 374.4 | 299.1 | 376.8 |
|  |  |  |  |  |  |  |
| Step 2 | 311.3 | 394.3 | 293.5 | 371.2 | 295.6 | 371.8 |
|  |  |  |  |  |  |  |
| Improvement | 3.6 | 5.7 | 4.4 | 6.2 | 3.2 | 5.0 |

Fig. S1. Distribution of maternal age

Fig. S2. Distribution of maternal height

Fig. S3. Distribution of maternal weight

Fig. S4. Distribution of maternal BMI

Fig. S5. Distribution of gravidity

Fig. S6. Distribution of parity

Fig. S7. Distribution of previous abortion times

Fig. S8. Distribution of GA

Fig. S9. Atmospheric SO_2_ concentration between January 2019 and December 2022 at the center monitoring station of the downtown
